# Cellular immune response of *Amblyomma sculptum* and *Amblyomma americanum* to entomopathogenic fungi: Implications for biological tick control

**DOI:** 10.1111/mve.70072

**Published:** 2026-04-10

**Authors:** Cárita de Souza Ribeiro‐Silva, Valesca Henrique Lima, Salorrane Miranda N. Pinto, Gustavo Felizardo S. Sandes, Victor Hugo Ribeiro Costa, Albert Mulenga, Adela S. Oliva Chavez, Éverton Kort Kamp Fernandes

**Affiliations:** ^1^ Instituto de Patologia Tropical e Saúde Pública Universidade Federal de Goiás Goiânia Brazil; ^2^ Escola de Veterinária e Zootecnia Universidade Federal de Goiás Goiânia Brazil; ^3^ Department of Veterinary Pathobiology College of Veterinary Medicine, Texas A&M University College Station Texas USA; ^4^ Department of Entomology Texas A&M University College Station Texas USA; ^5^ Present address: Department of Entomology University of Wisconsin‐Madison Madison Wisconsin USA

**Keywords:** *Beauveria bassiana*, haemocytes, *Metarhizium robertsii*, phenoloxidase, ticks

## Abstract

*Amblyomma* spp. (Acari, Ixodidae) serve as primary vectors for numerous pathogens affecting humans and animals. Unlike other arthropods, ixodid ticks exhibit notably less susceptibility to entomopathogenic fungi, which are widely used as biological control agents. This study aimed to determine whether the dynamics of the cellular immune response to fungal infection differ between *Amblyomma sculptum* and *Amblyomma americanum*. Engorged female ticks were treated with conidial suspensions of *Metarhizium robertsii* ARSEF 2575 or *Beauveria bassiana* ARSEF 9588. For the survival assay, ticks were immersed in suspensions of 5 × 10^8^ conidia mL^−1^. Inoculation with 5 × 10^7^ conidia mL^−1^ was used in assays to evaluate immune responses. Haemolymph was collected 24 h post‐treatment, and smears were prepared for microscopy‐based characterization of haemocyte responses. Confocal and fluorescence microscopy were used to classify, quantify and visualize the haemocyte types, including granulocytes, plasmatocytes, prohaemocytes, oenocytoids and spherulocytes. Our findings reveal that plasmatocytes dominate the haemocyte population in *A. sculptum*, whereas granulocytes are predominant in *A. americanum*, both showing reduced concentrations following *B. bassiana* exposure. Prohaemocytes and oenocytoids were present in all groups but at lower frequencies. Additionally, *B. bassiana* was more effective against *A. sculptum*, whereas *M. robertsii* exhibited greater efficacy against *A. americanum*, with phenoloxidase activity of ~29%. This study provides crucial insights into the cellular immune defences of *Amblyomma* ticks and highlights potential fungal strains that may overcome tick antifungal susceptibility. These findings establish a foundation for future research on haemocyte‐mediated immunity in ixodid ticks, a topic with limited existing literature and contribute to the development of targeted biological control strategies.

## Graphical abstract

- *Amblyomma sculptum* showed a plasmatocyte‐dominant haemocyte profile. *Beauveria bassiana* increased plasmatocytes and reduced granulocytes, whereas *Metarhizium robertsii* did not cause significant changes.
- *Amblyomma americanum* showed a granulocyte‐dominant haemocyte profile. *Beauveria bassiana* reduced total haemocytes and granulocytes. *B. bassiana* and *Metarhizium robertsii* infection induced phenoloxidase activity.
- Haemocyte profiles help explain tick antifungal tolerance and support targeted selection of entomopathogenic fungi for biological control strategies.

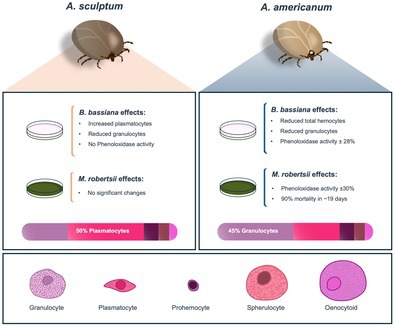

## INTRODUCTION

*Amblyomma* (Koch, 1844) tick species are important vectors of multiple human pathogens, including *Rickettsia* spp., the agents of Rocky Mountain Spotted Fever (Araújo et al., 2015), *Borrelia lonestari* (Stromdahl et al., 2003) and *Borrelia burgdorferi*, the etiological agent of Baggio‐Yoshinari syndrome (Lyme‐like disease) (Miziara et al., 2018). Furthermore, *Amblyomma* spp. transmit *Coxiella burnetii* (Q fever), *Borrelia recurrentis* (related to human relapsing fever), Crimean‐Congo haemorrhagic fever virus and *Ehrlichia chaffeensis* (which leads to human ehrlichiosis) (Damasceno & Guerra, 2018; Labruna & Mattar V, 2011; Massard & Fonseca, 2004; Rozental et al., 2002).

*Amblyomma* spp. are aggressive biters, and they are the primary tick species that bite humans in Brazil and the United States (Labruna et al., 2004; Mixson et al., 2006). Thus, these ticks represent an important threat to human health in a wide range of regions. Arthropod populations are controlled particularly by the use of chemical products, such as organophosphates and pyrethroids. These products, when used repeatedly and indiscriminately, may lead to environmental contamination and promote the selection of acaricide‐resistant ticks within populations. This issue has been well‐documented in *Rhipicephalus* spp. ticks in Mexico, Australia and Brazil (Barré et al., 2008; Ribeiro et al., 2010). Increasing reports of acaricide resistance or tolerance have been reported in *Amblyomma* spp., including *Amblyomma variegatum* in Africa (Evans et al., 2024), *Amblyomma sculptum* in Brazil (Cardoso et al., 2023) and *Amblyomma americanum* in the United States (Kaplan et al., 2022). The potential development of acaricide resistance in these tick populations has motivated studies on sustainable alternatives for pest control, including the use of biological control methods (Chandler et al., 2000).

Entomopathogenic fungi naturally infect ticks and have shown high virulence. Although several species exist in nature, the most investigated against ticks are *Metarhizium* spp. and *Beauveria* spp. (Fernandes & Bittencourt, 2008). These fungi are not only alternatives to chemical products but may also be used in combination with these chemicals in integrated pest management programs (Bahiense et al., 2006; Sousa et al., 2011). These microorganisms usually invade their hosts by penetrating directly through the cuticle (Hartelt et al., 2008). Once the fungi overcome this first barrier, the fungal hyphae invade the host haemocoel, where they encounter immune defences (Lavine & Strand, 2002). Tick responses to pathogens rely on innate immunity, which involves coordinated cellular and humoral mechanisms.

Haemocytes are the primary cells that respond to microorganism invasion by performing phagocytosis (Fiorotti et al., 2019), encapsulation and nodulation (Ceraul et al., 2002). These cells also modulate the production of reactive oxygen (Alberdi et al., 2019) and nitrogen species (Feitosa et al., 2018) and interact with humoral components, such as the complement system (Fiorotti et al., 2022) and the phenoloxidase cascade (Feitosa et al., 2018). Conserved signalling pathways modulate the gene expression of antimicrobial peptides, such as defensins (Szczotko et al., 2024) and microplusin (Martins et al., 2019), and other immune effectors, including lysozymes (Tanaka et al., 2010), and transferrins involved in oxidative stress responses (Solyman et al., 2021). Some pathogens, however, can manipulate the tick immune response, leading to the downregulation of key genes and effectors required to control infection (Alberdi et al., 2019; Rosa et al., 2016). These findings highlight the complexity of immune responses and host‐pathogen interactions.

Variations in susceptibility to entomopathogenic fungi have been reported among tick species and even within the same species at different developmental stages (Gindin et al., 2002; Ribeiro‐Silva et al., 2022). The tick cuticle is widely recognized as the primary barrier to fungal infection (Ribeiro‐Silva et al., 2022); however, certain fungi, including *Metarhizium* and *Beauveria* species, have demonstrated their ability to penetrate this barrier and infect ticks (Ment et al., 2012; Sullivan et al., 2022). Despite their ability to infect ticks, these fungi may cause lethality levels below the threshold required for effective biological tick control. Ticks are known to differ in immune cell populations among different species, and the prophenoloxidase (PPO) activating system is thought to be absent in *A. americanum*; however, it has been reported in *Rhipicephalus sanguineus* sensu lato (s.l.) and *Ornithodoros moubata* (Fogaça et al., 2021). This study aims to determine whether *A. sculptum* and *A. americanum* differ in their cellular immune response to fungal infection, with emphasis on haemocyte dynamics and phenoloxidase activity. We hypothesized that variation in cellular immune response, potentially shaped by their ecological backgrounds, could contribute to differences in susceptibility to entomopathogenic fungi. This information can be used to design in vitro tests and other evaluations that determine the best fungal strains for controlling ticks.

## MATERIALS AND METHODS

### Fungal culture and preparation of conidial suspensions

*Metarhizium robertsii* ARSEF 2575 and *Beauveria bassiana* ARSEF 9588 were obtained from the USDA‐ARS Collection of Entomopathogenic Fungal Cultures (ARSEF), Ithaca, NY, USA. These fungal isolates were cultured on 20 mL potato dextrose agar, supplemented with 1.0 g L^−1^ yeast extract (PDAY) (Bacto™, Difco Laboratories, Sparks, MD, Miami, FL, USA) in Petri plates (100 × 15 mm) at 27 ± 1°C and a 12:12 h light:dark (L:D) photoperiod for 15 days. Aerial conidia were harvested from sporulated cultures using a spatula, suspended in a sterile polyethylene sorbitan monooleate (Tween 80®, Sigma‐Aldrich, St. Louis, MO, USA) aqueous solution (0.01%, v/v), and then adjusted to 5 × 10^7^ conidia mL^−1^ and 5 × 10^8^ conidia mL^−1^ prior to tick treatments.

### Ticks: fungal inoculation and haemolymph collection

Engorged females of *A. sculptum* were collected from naturally infested horses that had not been treated with acaricides for at least 30 days. The horses were obtained from the School of Veterinary and Animal Science of the Federal University of Goiás, and from animals rescued by the Zoonosis Control Center of Goiânia, Brazil (CEUA/UFG permit: 055/2021).

*A. americanum* females were obtained from a certified pathogen‐free colony maintained at Oklahoma State University, USA. These ticks were infested onto New Zealand White rabbits purchased from Charles River Laboratories (Wilmington, Massachusetts, USA). Ticks were fed for 20 days inside Ethylene Vinyl Acetate (EVA) foam capsules as previously described (Leal‐Galvan et al., 2022) and according to the procedures outlined in the Animal Usage Protocol (AUP) #2020–0026, approved by the Institutional Animal Care and Use Committee (IACUC) of Texas A&M University.

Engorged females were sanitized in a 1% bleach solution for 3 min, rinsed in sterile distilled water and dried on sterile paper towels. Females from each species were then separated into three groups, with each group consisting of five ticks: (1) the control group treated with 0.01% (v/v) Tween® 80 solution; (2) the group treated with a conidial suspension of *B. bassiana*; and (3) the group treated with a conidial suspension of *M. robertsii*. Treatment was performed by inoculating 5 μL of conidial suspension, at 5 × 10^7^ conidia mL^−1^, into the foramen located between the dorsal scutum and the capitulum, using an insulin needle (0.3 mm). After treatment, the engorged females were incubated at 27 ± 1°C and relative humidity (RH) >90% for 24 h. The tick's cuticle was pierced with a 0.3‐mm needle, and then a gentle pressure was exerted on the tick's body to collect haemolymph samples using 0.3‐mm glass microcapillary tubes. The collected haemolymph was used for microscopy analysis and phenoloxidase activity assays.

### Quantification and characterization of haemocytes

A 10‐μL aliquot of total haemocytes suspended in 0.01 M phosphate‐buffered saline (PBS) at pH 7.2 (1:1) was immediately quantified in a haemocytometer using a light microscope (Olympus BX43F and Nikon Eclipse E200) at 400× magnification. A sample of haemolymph was also deposited on glass slides to perform a smear. The slides were stained using a three‐step stain set (Richard‐Allan Scientific™) according to the manufacturer's specifications and analysed for morphological characterization of haemocytes at 400× magnification using a light microscope. Haemocytes from *A. americanum* were visualized using an Olympus model BX43F (Shinjuku City, Tokyo, Japan) at Texas A&M University. Those from *A. sculptum* were visualized using a Nikon Eclipse model E200 (Nikon Instruments Inc., Tokyo, Japan) at the Federal University of Goiás.

Three slides were prepared for each treatment group, with a total of nine slides after the third repetition of the experiment. On each slide, 100 cells were randomly counted and morphologically classified based on cell size and shape, nuclear size and location, presence of granules or vacuoles and cytoplasm homogeneity. The mean and the standard deviation of the cell types from the treatment and control groups were calculated.

### Confocal fluorescence microscopy

Haemolymph from *A. americanum* was collected, and new smears were prepared and held in a humid chamber for 30 min to allow the cells to adhere to the glass slide. The smears were then fixed with 4% paraformaldehyde in PBS (Thermo Fisher Scientific Inc.) for 10 min. Cell permeabilization was performed by washing slides with PBS buffer containing 0.2% (v/v) Tween 20®. The nuclei were stained in blue with 4′‐6‐diamidino‐2 phenylindole (DAPI) (Invitrogen, Thermo Fisher Scientific Inc.) for 1 h, at room temperature (RT), avoiding light. Phalloidin fluorescein isothiocyanate (Abnova Corporation, Taiwan) was used to stain in green the actin filaments and cell membrane proteins for 30 min at RT. Fluorescence signals were confirmed in a dark room, as described by Blanco et al. (2017), with modifications. Cells were visualized using an LSM‐780 Confocal Microscope (Zeiss, Germany) at Texas A&M Veterinary Medicine & Biomedical Sciences. Images were acquired with an Airyscan superresolution detector (32‐channel GaAsP‐PMT). Fluorescence microscopy images were used as complementary tools to visualize cell morphological traits and were not used as primary evidence for haemocyte characterization.

### Phenoloxidase activity assay

Two microliters of haemolymph were collected from each tick (*A. sculptum* and *A. americanum*) from treatment or control groups. These samples were then incubated for 10 min with 28 μL of 0.01 M Cacodylate buffer containing 0.0005 M CaCl_2_ at pH 7.0 in 96‐well cell culture plates. Ten microliters of a saturated solution of L‐Dopamine (Sigma Aldrich, USA) at 4 mg mL^−1^ were added to the mixture and held for an additional 20 min at room temperature, according to the protocol developed by Ling and Yu (2005). The ELISA 96‐well plate reader measured absorbance at 490 nm, using cacodylate buffer as the blank, as described by Mello et al. (1995).

Enzyme activity is expressed as a percentage, calculated based on the activation observed in the group treated with trypsin (1 mg mL^−1^ of haemolymph during a 30‐min incubation), which serves as a reference for determining the activation levels in the other treatments. Three replicates were performed using both tick species and different fungal cultures, with samples read in triplicate for each evaluation.

### Assays with unfed adult ticks—Survival analysis

Unfed females of *A. sculptum* or *A. americanum* were separated into three groups of five. Females were immersed in conidial suspension (5 × 10^8^ conidia mL^−1^) of *B. bassiana* ARSEF 9588 or *M. robertsii* ARSEF 2575 for 3 min, whereas the control group was submerged in 0.01% Tween 80®. After exposure, ticks were incubated at 27 ± 1°C and RH ≥90% and evaluated daily for 19 days to determine survival among different treatments. The entire experiment was performed three times.

### Data analysis

Data sets were verified for homoscedasticity using the Bartlett test and normality using the Shapiro‐Wilk test. The mean values of phenoloxidase activity and haemocyte characterization were then submitted to Kruskal‐Wallis, followed by Dunn's test for multiple comparisons and *p*‐values were adjusted with the Benjamini‐Hochberg correction, using the ‘easyanova’ package in R software (R Core Team 2016). GraphPad Prism version 10.4.1 was used to analyse the mean of haemocyte counts using analysis of variance (ANOVA) followed by Dunnett's test. Survival curves were constructed in GraphPad Prism and analysed using the Log‐rank (Mantel‐Cox) test. The mean values for phenoloxidase activity and haemocyte characterization were imported into GraphPad Prism to create the graphs. Values of *p* less than 0.05 were significant (Arnhold, 2013). Adobe Illustrator 2023, version 27.6.1, was used for the overall design and layout of the images.

## RESULTS

### Quantification and characterization of haemocytes

**FIGURE 1:**
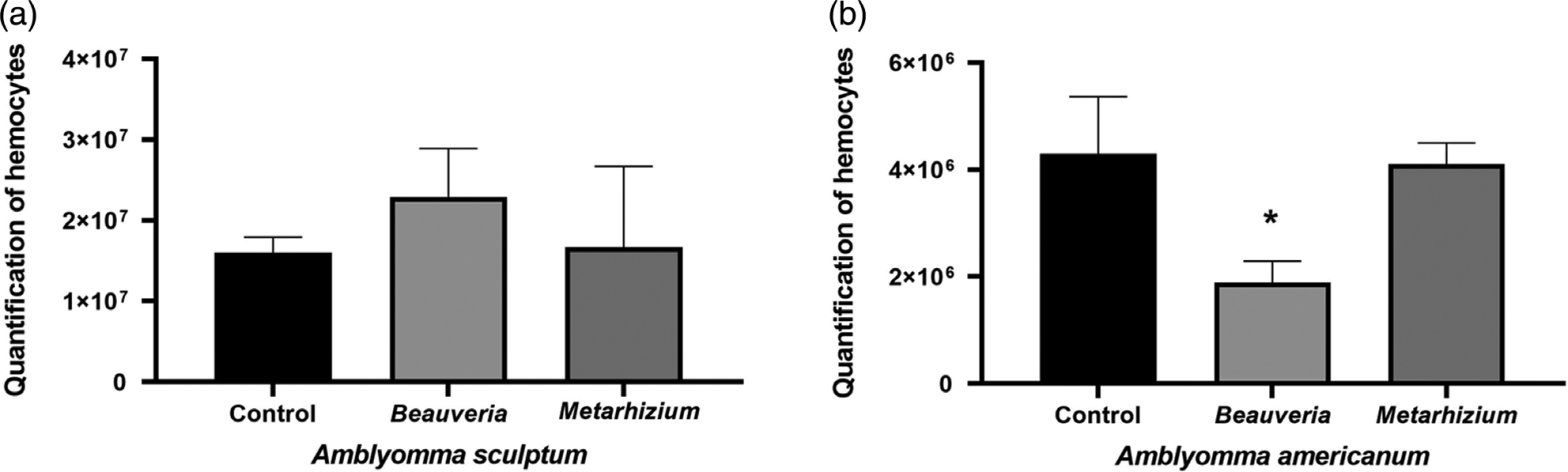
Total number of circulating haemocytes in the haemolymph of *Amblyomma sculptum* (a) or *Amblyomma americanum* (b) 24 h after treatment with entomopathogenic fungi: *Metarhizium robertsii* ARSEF 2575 (A 2575) or *Beauveria bassiana* ARSEF 9588 (A 9588). (_*_) indicates a significant difference (*p* < 0.05) in the number of haemocytes, within the same tick species, by analysis of variance (ANOVA) followed by Dunnett's test.

The total number of circulating haemocytes in the haemolymph of *A. sculptum* (± 1 × 10^7^ cells mL^−1^) appears to be greater than that found in *A. americanum* (± 4 × 10^6^ cells mL^−1^) in non‐challenged ticks (Figure 1). However, no statistical comparisons between species were conducted, as the analysis focused on differences within each species and experiments were conducted at different locations and times, which could introduce variations that might obscure the results. Further, *B. bassiana* infection significantly reduced the number of circulating haemocytes in *A. americanum* haemolymph (*F*_(2,6)_ = 4.85, *p* adjusted = 0.0443) (Figure 1b). This response was not observed in *A. sculptum*, which did not show a significant change in haemocyte numbers despite the treatment with *B. bassiana* (Figure 1a). *M. robertsii* did not induce significant changes in the number of circulating haemocytes (Figure 1a,b).

**FIGURE 2:**
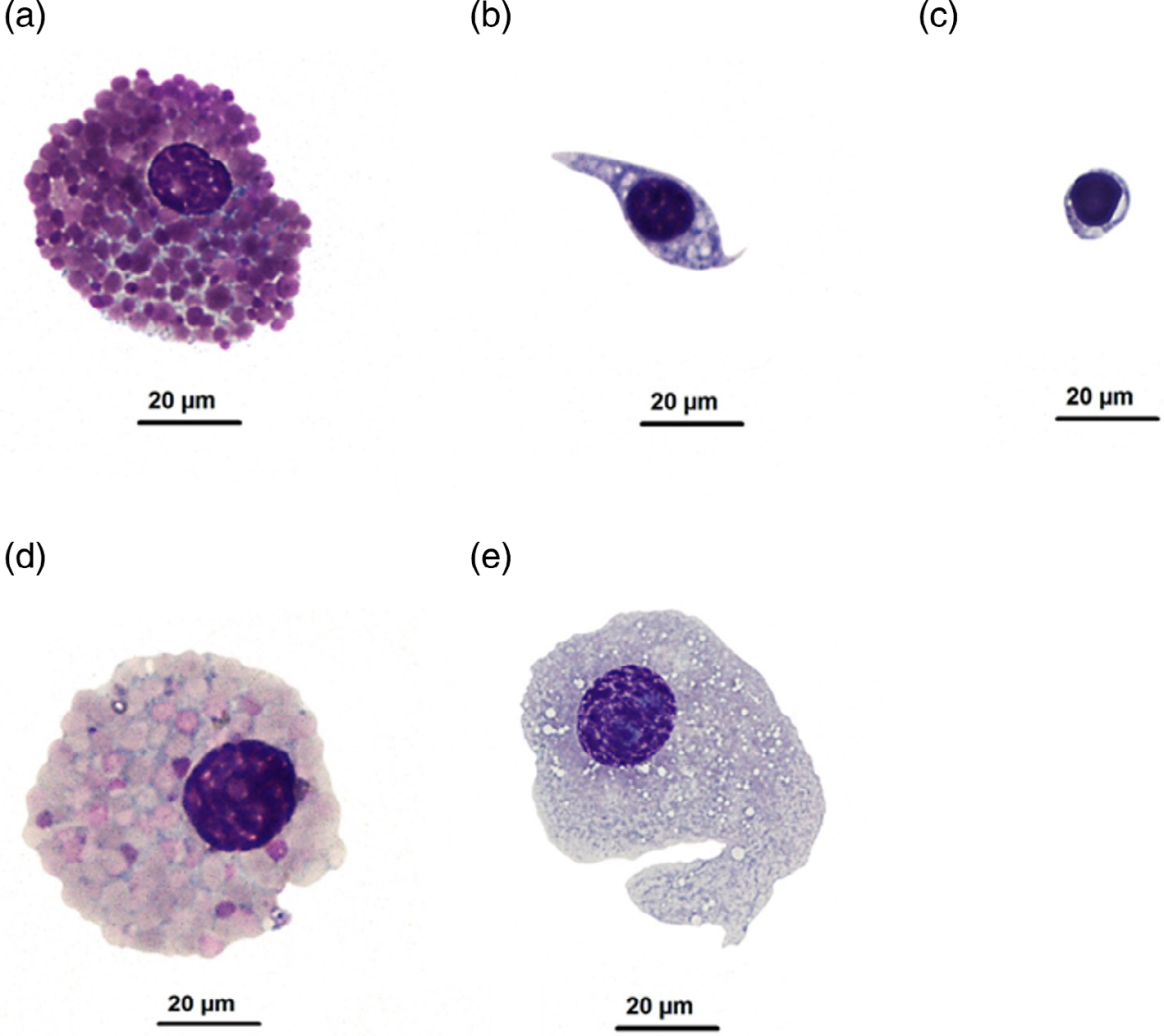
Types of haemocytes in the haemolymph of *Amblyomma sculptum*. The cells were stained using the TreeStep kit and analysed at 1000× magnification under a light microscope equipped with a digital camera: Granulocyte (a); plasmatocyte (b); prohaemocyte (c); spherulocyte (d); oenocytoid (e). Scale bar: 20 μm.

**FIGURE 3:**
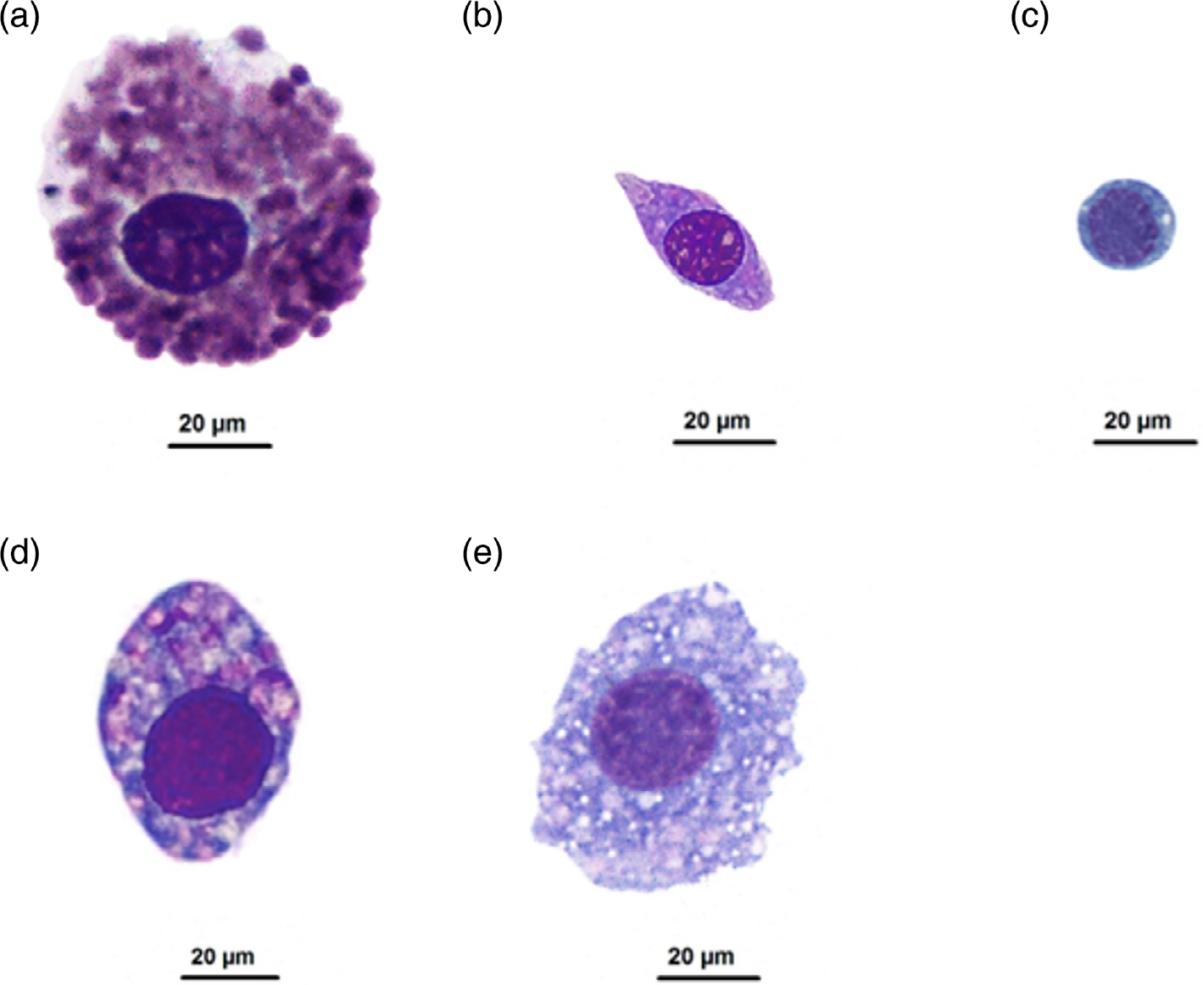
Types of haemocytes in the haemolymph of *Amblyomma americanum*. The cells were stained using the TreeStep kit and analysed at 1000× magnification under a light microscope equipped with a digital camera: Granulocyte (a); plasmatocyte (b), prohaemocyte (c); spherulocyte (d); oenocytoid (e). Scale bar: 20 μm.

**FIGURE 4:**
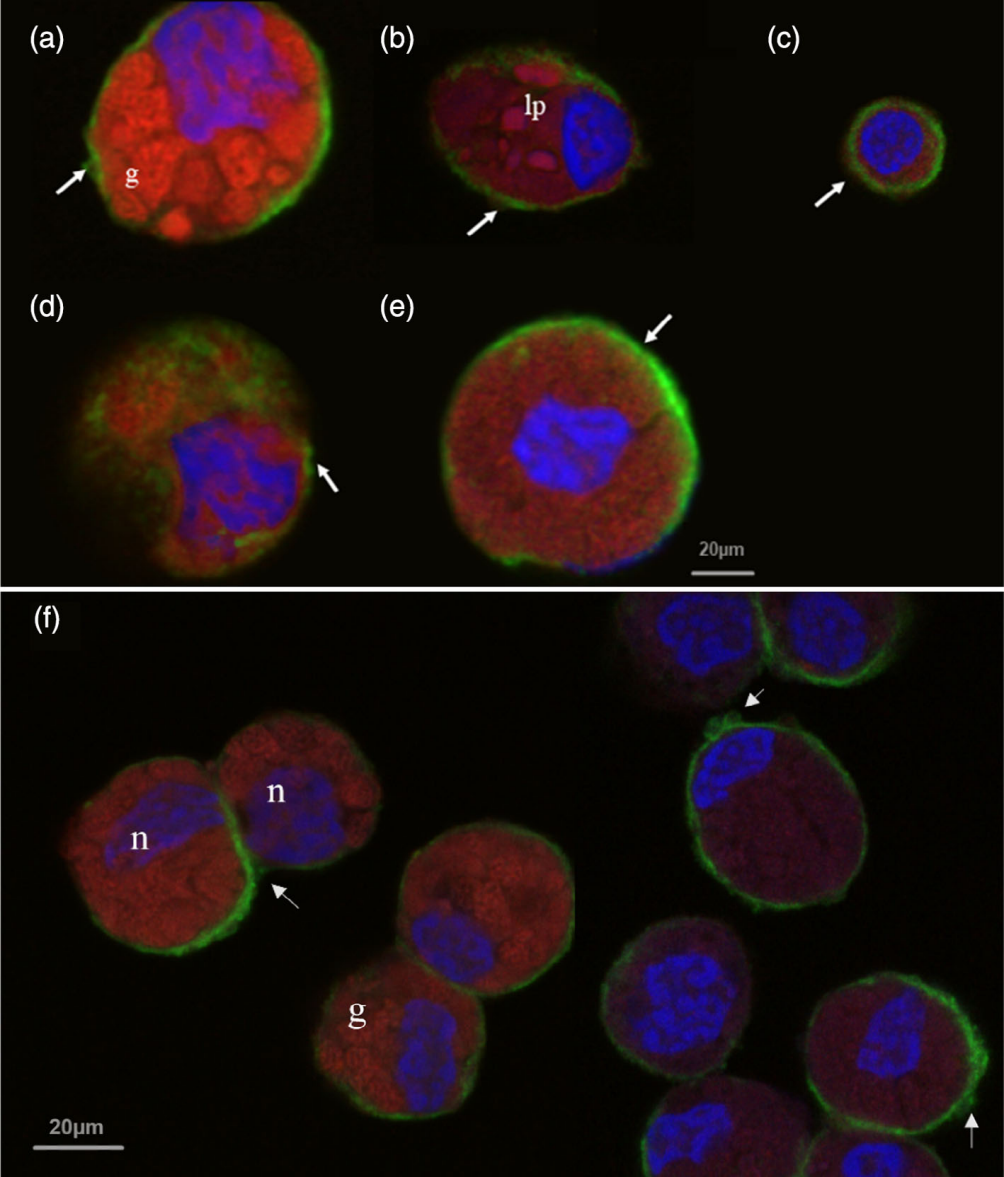
Confocal fluorescence microscopy of circulating haemocytes from *Amblyomma americanum* ticks without fungal treatment, visualized at 1000 × magnification. Nuclei were stained blue with DAPI (4′,6‐diamidino‐2‐phenylindole), and cell membranes were stained green with phalloidin fluorescein isothiocyanate. The red autofluorescence in the cytoplasm likely reflects the biochemical composition of cellular components. We hypothesize that the intense red signal corresponds to cytoplasmic granules, whereas the less intense fluorescence reflects a more homogeneous cytoplasmic content. Based on cell size and cytoplasmic fluorescence, the haemocytes shown in this figure may represent: Granulocyte (a); plasmatocyte (b); prohaemocyte (c); spherulocyte (d); oenocytoid (e). Grouped haemocytes in the haemolymph are also shown (f). Arrows indicate the cell membranes; (n) nuclei; (g) granules; and (lp) lipid inclusions. Scale bars: 20 μm.

Haemolymph from *A. sculptum* engorged females ranged in colour from translucent to blue (not shown). *A. americanum* haemolymph presented a translucent appearance. Five cell types were found in both tick species: Granulocytes, Plasmatocytes, Prohaemocytes, Spherulocytes and Oenocytoids (Figures 2 and 3). Notably, some granulocyte cells were found clumping together after infection with entomopathogenic fungi (Figure 4). Immune cells showed the following morphological characteristics: (1) Granulocytes: The cells were round or oval with many eosinophilic granules in the cytoplasm, which may have been covering the nuclei (Figures 2a and 3a). While two distinct granulocyte types were detected in *A. sculptum*, we did not conduct granule‐specific analyses and therefore treated them as a single category during analysis. Type II granulocytes are generally larger than 16 μm and have granules that cover the nucleus. The average size of this cell type was approximately 25 μm. (2) Plasmatocytes: These were polymorphic cells with an average size of around 20 μm despite their wide variation in shape. Plasmatocyte cells have a central or eccentric nucleus, vacuoles and few granules in the cytoplasm (Figures 2b and 3b). This cell type was distinguished by its cytoplasm, which had projections. (3) Prohaemocytes: They were the smallest cell type observed, with a very close nucleus‐to‐cytoplasm ratio, a size of approximately 14 μm and a central nucleus (Figures 2c and 3c). (4) Spherulocytes: They were cells with an oval or circular shape and a nucleus in an eccentric position. Their size was approximately 23 μm. The cytoplasm contained abundant spherules that showed minimal staining with the dye (Figures 2d and 3d). (5) Oenocytoids: These cells had small granules with irregular cytoplasmic margins, a spongy and refractive appearance, and an eccentric nucleus. They were large cells (around 50 μm) and rarely present in the haemolymph of both tick species investigated in this study (Figures 2e and 3e).

### Differences in haemocyte counts

**FIGURE 5:**
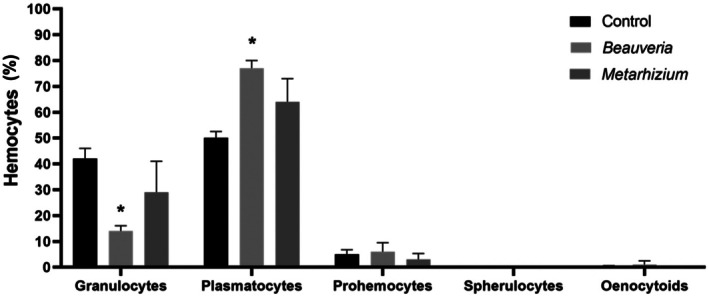
Percentage of haemocytes in the haemolymph of *Amblyomma sculptum* 24 h after treatment with entomopathogenic fungi: *Metarhizium robertsii* ARSEF 2575 (A 2575) or *Beauveria bassiana* ARSEF 9588 (A 9588). (_*_) indicates a significant difference (*p* < 0.05), within the same haemocyte type, by the Kruskal‐Wallis test followed by Dunn's multiple comparisons with Benjamini‐Hochberg correction.

**FIGURE 6:**
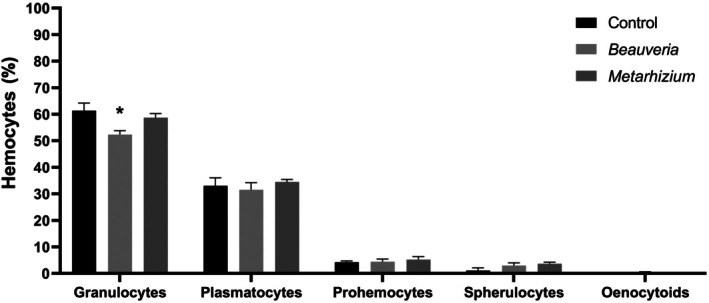
Percentage of haemocytes in the haemolymph of *Amblyomma americanum* 24 h after treatment with entomopathogenic fungi: *Metarhizium robertsii* ARSEF 2575 (A 2575) or *Beauveria bassiana* ARSEF 9588 (A 9588). (_*_) Indicates a significant difference (*p* < 0.05) within the same haemocyte type, by Kruskal‐Wallis test followed by Dunn's multiple comparisons with Benjamini‐Hochberg correction.

In *A. sculptum*, plasmatocytes were the predominant cell type, comprising approximately 50% of the cells found in their haemolymph (Figure 5). The infection with *B. bassiana* resulted in a significant increase in the number of plasmatocytes when compared to the control group (*χ* ^2^ = 7.2, *p =* 0.027, *p* adjusted = 0.022). Granulocytes were the second most abundant cell type in uninfected *A. sculptum* but were significantly reduced in ticks infected with *B. bassiana* (Figure 5; *χ* ^2^ = 7.2, *p =* 0.027, *p* adjusted *=* 0.022). Prohaemocytes (*χ* ^2^ = 5.66, *p =* 0.059), spherulocytes (*χ* ^2^ = 1.23, *p =* 0.54) and oenocytoids (*χ* ^2^ = 4.49, *p =* 0.1) were found in low numbers and were not affected by infection with either entomopathogenic fungi. In *A. americanum*, granulocytes were the most abundant cells, followed by plasmatocytes. The infection with *B. bassiana* resulted in a reduced number of circulating granulocytes (Figure 6; *χ* ^2^ = 6.0, *p =* 0.049, *p* adjusted = 0.049), whereas the mean number of the other cell types remained unchanged.

### Confocal fluorescence microscopy

To further visualize the morphology of haemocytes, we also used confocal microscopy (Figure 4). The abundant granules in the granulocytes were highlighted in red (Figure 4a). Additionally, the cellular lipid inclusions (lp) on plasmatocytes (Figure 4b), the low proportion of cytoplasm and nucleus of prohaemocytes (Figure 4c) and the typical refraction of spherulocytes (Figure 4d) were clearly detectable.

### Differences in phenoloxidase activity following fungal treatment

Phenoloxidase activity was not detected in adult *A. sculptum*, regardless of treatment. However, *A. americanum* phenoloxidase activity was detected and increased significantly when ticks were infected with *B. bassiana* (by approximately 28%; *χ* ^2^ = 10.9, *p =* 0.004, *p* adjusted = 0.012) or *M. robertsii* (approximately 30%; *χ* ^2^ = 10.9, *p =* 0.004, *p* adjusted = 0.007).

### Differences in tick survival following fungal treatment

**FIGURE 7:**
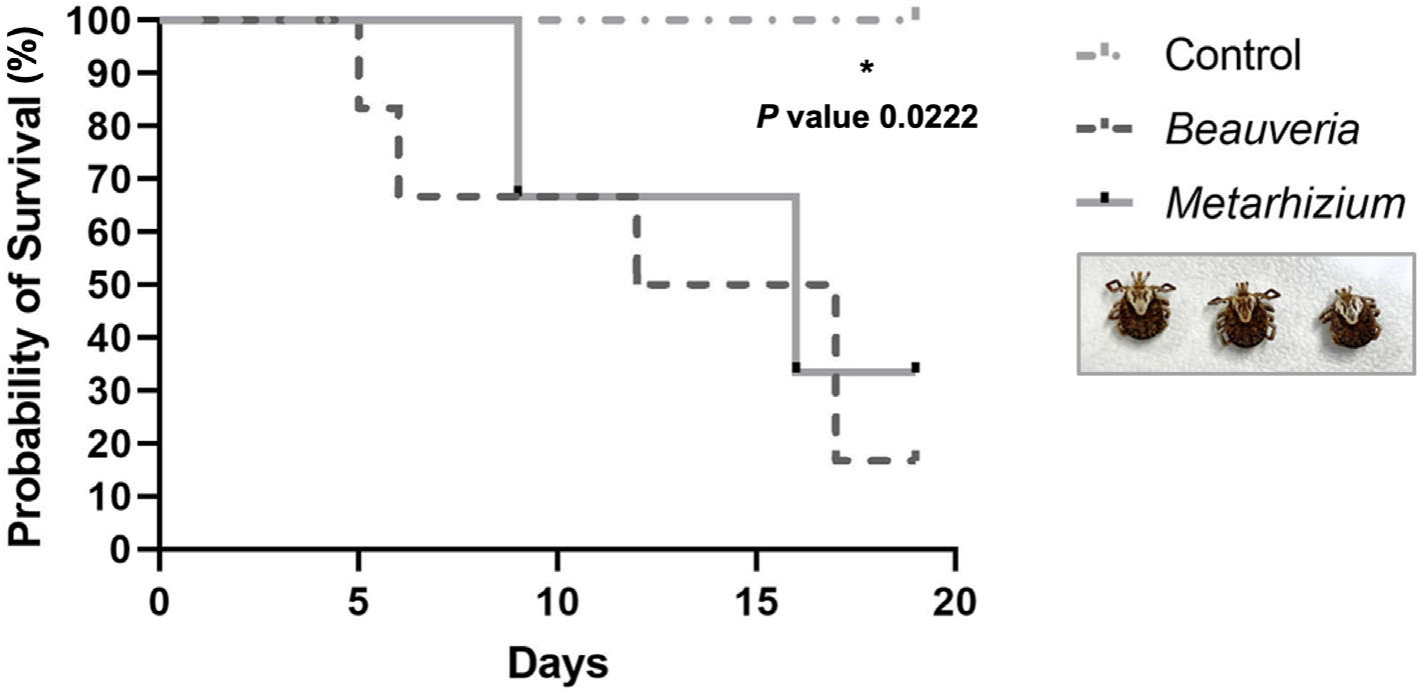
Probability of survival of *Amblyomma sculptum* unfed females in relation to the time after infection with *Beauveria bassiana* ARSEF 9588 (A 9588) or *Metarhizium robertsii* ARSEF 2575 (A 2575) by the Log‐Rank (Mantel‐Cox) test (*p* = 0.0222).

**FIGURE 8:**
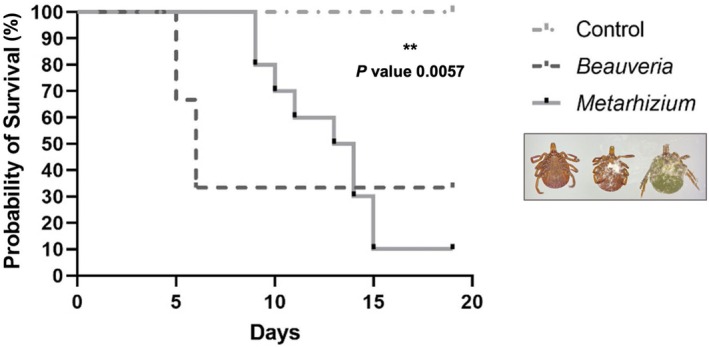
Probability of survival of *Amblyomma americanum* unfed females in relation to the time after infection with *Beauveria bassiana* ARSEF 9588 (A 9588) or *Metarhizium robertsii* ARSEF 2575 (A 2575) by the Log‐Rank (Mantel‐Cox) test (*p* = 0.0057).

Upon treatment with a fungal suspension, unfed female *A. sculptum* ticks required more than 12 days for the survival curve to begin declining by half when exposed to *B. bassiana* and at least 16 days when exposed to *M. robertsii* (Figure 7; *χ* ^2^ = 7.618, *df* = 2, *p =* 0.0222). However, *A. americanum* treated with *B. bassiana* exhibited a reduction in survival probability within 6 days post‐treatment. In contrast, *M. robertsii* treatment caused a gradual decline, with approximately 90% mortality reached by day 15 post‐treatment (Figure 8; *χ* ^2^ = 10.32, *df* = 2, *p =* 0.0057).

## DISCUSSION

The development of control strategies for ixodid ticks requires an understanding of their immune system. The haemolymph cells in arthropods are nucleated and perform four essential functions, including phagocytosis, nutrient storage, nodulation and encapsulation, in addition to mediating the humoral response (Fiorotti et al., 2019; Lavine & Strand, 2002; Sonenshine & Hynes, 2008; Strand, 2008). The defence mechanism of ixodids, however, still raises many reservations and disagreements (Fogaça et al., 2021). Plasmatocytes, granulocytes and prohaemocytes are the three main types of haemocytes frequently reported in ticks, although different species may exhibit additional cell types. Several studies propose classifications of haemocytes in tick haemolymph, often comparing them to those found in other arthropods, particularly insects (Feitosa et al., 2015; Fogaça et al., 2021).

Five main types of haemocytes have been identified by ultrastructural characterization in *Amblyomma* ticks: granulocytes, plasmatocytes, prohaemocytes, oenocytoids and spherulocytes. These cell types appear to be conserved across different species within the genus *Amblyomma*, as reported in recent studies (Adegoke et al., 2023; Adegoke, Hanson, et al., 2024; Reis et al., 2024). In addition to these morphologically defined cell types, sequencing analysis has revealed greater cellular diversity, identifying 14 haemocyte populations in *A. americanum* (Adegoke, Ribeiro, et al., 2024). Despite this conserved classification, the relative abundance and dynamics of haemocytes may vary under physiological conditions, such as changes in feeding status or exposure to pathogens. In the present study, we identified and described the same five immune cell types in the haemolymph of both *A. sculptum* and *A. americanum*.

Plasmatocytes were the most abundant cells in the haemolymph of *A. sculptum* engorged females, corresponding to approximately 50% of the haemocytes, whereas in *A. americanum*, this cell type was the second most abundant. Many factors can alter the cellular composition of tick haemolymph, including microbial infections (Fisher & Ganesalingam, 1970). Following the infection with *B. bassiana*, changes were observed in the total haemocyte counts of *A. americanum* and *A. sculptum*. A previous study using *Rhipicephalus microplus* reported a fivefold increase in the total number of haemocytes 48 h after treatment with *B. bassiana* (Freitas et al., 2015). This finding contrasts with a study by Fiorotti et al. (2018), which demonstrated that the number of haemocytes decreased following 24 h after inoculation with *M. robertsii* ARSEF 2575. However, the results of the present study demonstrated that the number of circulating plasmatocytes increased 24 h after treatment with *B. bassiana* ARSEF 9588. Morphologically, plasmatocytes were the most distinct cell type in *A. sculptum* and *A. americanum*, characterized by cytoplasmic projections and eccentric or central nuclei. The cell size ranged around 20 μm, like those found in *A. sculptum* by Reis et al. (2024). Furthermore, the morphology of the plasmatocytes described in this study is similar to that identified in *R. microplus* (Fiorotti et al., 2019; Reis et al., 2024).

The most abundant cells in the haemolymph of *A. americanum* were granulocytes. After exposure to *B. bassiana*, their numbers decreased, whereas no reduction was observed following treatment with *M. robertsii*. In *A. sculptum*, circulating granulocytes also decreased, showing loss of cytoplasmic granules, as previously reported for the cattle tick, *R. microplus* (Fiorotti et al., 2019). Granulocytes are recruited for immune processes such as phagocytosis and encapsulation (Sonenshine & Hynes, 2008), and their reduction in the haemolymph during fungal infection may reflect active recruitment to tissues as a defence mechanism (Gunnarsson, 1988; King & Hillyer, 2012). Besides tissue recruitment, the production of toxic metabolites by *B. bassiana* can suppress host immunity (Valencia et al., 2011; Zibaee et al., 2011), and damage to haemocyte membranes has been reported in arthropods following *B. bassiana* infection (Fan et al., 2025). Furthermore, immune stress induced by an entomopathogenic fungus may contribute to a reduction in haemocytes through apoptosis or necrosis (Wrońska et al., 2022).

In our study, granulocytes were observed adhering to one another under microscopy, indicating haemocyte aggregation, a phenomenon previously reported by Cho and Cho (2019). These researchers also described granulocytes actively producing amoeba‐like filaments and extracellular traps from their plasma membranes to gather other haemocytes and carry out cellular immune responses in *Gryllus bimaculatus*. Morphologically, granulocytes displayed irregularly shaped cytoplasmic granules, some of which showed signs of degradation. Additionally, the cell size was approximately 25 μm, similar to that reported for *A. sculptum* (22 μm; Reis et al., 2024), but smaller than that reported for *Rhipicephalus* spp. (Fiorotti et al., 2019).

The number of prohaemocytes was similar in both *Amblyomma* species, regardless of treatment. However, it is worth noting that this cell type is the precursor of other cell types, such as plasmatocytes and granulocytes, which were abundantly reported in the current study. This suggests that prohaemocytes may contribute to increasing and maintaining the number of circulating haemocytes after an immune challenge (Feitosa et al., 2015; Sonenshine & Roe, 2014). After fungal treatment, some small plasmatocytes with few projections were detected, possibly indicating recent cell differentiation. Prohaemocytes have a close ratio of nuclei to cytoplasm. They are small cells (±14 μm) like those found in *R. sanguineus* s.l., *R. microplus, A. sculptum* and *Dermacentor* sp. (Brinton & Burgdorfer, 1971; Carneiro & Daemon, 2002; Fiorotti et al., 2019; Reis et al., 2024).

Oenocytoid cells were not detected in significant numbers, with low counts in both *Amblyomma* species, regardless of the fungal infection. The absence of this cell type is suggested for some tick species under certain conditions, such as a pathogen infection (Feitosa et al., 2015; Fiorotti et al., 2019; Silva et al., 2006), and it seems not to be involved in phagocytosis processes but may be related to encapsulation mechanisms (Eggenberger et al., 1990; Fogaça et al., 2021). The oenocytoids are the largest haemocytes identified in this study, with an average size of approximately 50 μm. Although some studies report variations in size and internal composition, such as differences in mitochondria, it is generally agreed that oenocytoids are among the largest haemocytes (Carneiro & Daemon, 2002; Giulianini et al., 2003; Reis et al., 2024). Under light microscopy, the cytoplasmic details were unclear due to its spongy aspect, and the cell nucleus was eccentrically positioned.

Spherulocytes were rarely observed in *Amblyomma* spp., and no significant variation was observed even after treatment with entomopathogenic fungi. Silva et al. (2006) demonstrated that spherulocytes underwent cellular modifications after treatment with *Metarhizium* sp., making their precise identification difficult. Fiorotti et al. (2019) also reported the absence of spherulocytes in the haemolymph of infected *R. microplus*. The function of these haemocytes in ticks remains unclear, despite their reported presence in other arthropods. Their low numbers or absence of ticks, depending on the species, suggests they may not be directly involved in cell‐mediated arthropod defence (Brinton & Burgdorfer, 1971; Carneiro & Daemon, 2002; Feitosa et al., 2015; Fiorotti et al., 2019; Reis et al., 2024).

This study has identified, in both tick species, a few cells that resemble adipohaemocytes, previously described in *R. sanguineus* s.l. and in some species of the *Amblyomma cajennense* complex (Carneiro & Daemon, 2002; Feitosa et al., 2015; Fiorotti et al., 2019; Silva et al., 2006). Their identification was challenging because they were consistently found in low quantities. A study conducted by Hillyer et al. (2003) suggests a potential misidentification of this cell type, as it could be mistaken for fat body cells and thus overlooked. Additionally, recent research (Reis et al., 2024) suggests that this specific cell type is absent from the haemolymph of *A. sculptum*. Nevertheless, these cells may not be directly involved in responding to microorganisms. The adipohaemocyte‐like cells display an irregular shape and measure approximately 32 μm. They exhibit dense vesicles in their cytoplasm, along with fat vesicles. Each haemocyte type possesses distinct characteristics and may change in response to infections by microorganisms such as entomopathogenic fungi. This can occur through immunological processes, such as phagocytosis, or through secondary metabolites produced by fungi during infection (Fiorotti et al., 2019; Lavine & Strand, 2002; Wiegand et al., 2000). However, studies reporting the identification of haemocytes with phenoloxidase activity in ticks are scarce.

In some arthropods, cells such as granulocytes, plasmatocytes, spherulocytes and oenocytoids are associated with phenoloxidase activity. This activity helps to fight infections and can act as an opsonin, which is effective for the deposition of melanin (Ling & Yu, 2005; Lu et al., 2014; Lu & St. Leger, 2016). Although phenoloxidase plays an important role in the arthropod immune response, its activity has not been fully characterized in ticks. Our evaluations focused on the early immune response at 24 h post‐inoculation; however, longer exposure to pathogens could potentially alter the patterns of phenoloxidase activity and cellular responses. In *R. sanguineus*, phenoloxidase activity increased 5 days after infection with *Leishmania infantum*, whereas no activity was detected at earlier time points post‐infection (Feitosa et al., 2018), suggesting a delayed response. In *R*. *microplus* engorged females, inoculation with *M. anisopliae* caused a decrease in phenoloxidase activity only after 48 h post‐inoculation, but not at 24 or 72 h (Corrêa et al., 2021). These findings indicate that phenoloxidase activity in ticks may vary depending on the pathogen to which they are exposed and the tick species, highlighting the complexity of their immune regulation.

In the current study, *A. americanum*, but not *A. sculptum*, demonstrated increased phenoloxidase activity when infected with any of the entomopathogenic fungi tested. Despite this, the cellular response of *A. americanum* was insufficient to oppose the infection caused by *M. robertsii*. *A. sculptum* was more sensitive to infection by *B. bassiana* than by *M. robertsii*; however, long periods were required to reduce tick survival. It is essential to acknowledge that phenoloxidase activity constitutes only one component of the humoral immune response. Other mechanisms, such as antimicrobial peptides and reactive oxygen species (Fogaça et al., 2021), also play important roles in tick defence against pathogens; however, these were not assessed in this study. These results reinforce the importance of conducting studies that compare multiple aspects of the immune response and focus on each target species to enhance our understanding of the defence mechanisms of ixodid ticks.

Moreover, it is important to emphasize that the ticks used in this study originated from different sources: *A. sculptum* females were collected from naturally infested horses in Brazil and may have been previously exposed to environmental microorganisms, whereas *A. americanum* specimens were obtained from a pathogen‐free laboratory colony in the United States. This distinction is relevant, as prior microbial exposure may modulate the basal immune responses of ticks and contribute to some of the interspecific differences reported. These aspects should be considered in future studies evaluating the use of entomopathogenic fungi as biological control agents.

Although our results should be interpreted with caution, as naturally occurring microorganisms may have influenced tick immune responses, the modulation of haemocytes observed in *A. sculptum* and *A. americanum* supports our hypothesis that variation in cellular immunity contributes to differences in susceptibility to fungal infection. Given that haemocytes play a central role in fungal recognition and elimination, our findings provide valuable insights into host‐pathogen compatibility. Integrating haemocyte response profiles into laboratory assays may improve the selection of fungal isolates with enhanced infectivity or immune evasion capacity, thereby increasing the precision of biological control strategies targeting different tick species.

## AUTHOR CONTRIBUTIONS

**Cárita de Souza Ribeiro‐Silva**: Conceptualization; investigation; methodology; formal analysis; validation; writing—original draft; data curation; visualization; writing—review and editing. **Valesca Henrique Lima:** Investigation; methodology; formal analysis; validation; writing—original draft; data curation; visualization; writing—review and editing. **Salorrane Miranda N. Pinto:** Investigation; methodology; writing—original draft; formal analysis; validation; data curation; visualization; writing—review and editing. **Gustavo Felizardo S. Sandes:** Investigation; methodology; writing—review and editing. **Victor Hugo Ribeiro Costa:** Investigation; methodology; writing—review and editing. **Albert Mulenga:** Funding acquisition; validation; visualization; writing—review and editing. **Adela S. Oliva Chavez:** Conceptualization; funding acquisition; formal analysis; validation; writing—original draft; visualization; resources; supervision; writing—review and editing. **Éverton Kort Kamp Fernandes:** Conceptualization; funding acquisition; formal analysis; validation; writing—original draft; visualization; resources; supervision; project administration; writing—review and editing.

## FUNDING INFORMATION

This research was supported by grants from the Conselho Nacional de Desenvolvimento Científico e Tecnológico (CNPq) of Brazil (431928/2016–9, 420413/2023–5), the Instituto Nacional de Ciência e Tecnologia em Entomologia Molecular, Universidade Federal do Rio de Janeiro, Rio de Janeiro, Brazil (INCT‐EM, CNPq 465678/2014–9), and the Instituto Nacional de Ciência e Tecnologia em Bioinsumos Inovadores (INCT, CNPq 406803/2022–6). This research was also supported by a grant from the USDA Animal Health and Disease Research (A.H.D.R.) Project #TEX0‐9902 to A.O.C. CNPq provided the PQ 306319/2018–7 grant for É.K.K.F., a PhD scholarship for V.H.L and undergraduate research scholarships to G.F.S.S. and V.H.R.C. The Coordenação de Aperfeiçoamento de Pessoal de Nível Superior (CAPES) of Brazil provided PhD scholarships for C.S.R‐S., S.M.N.P. and V.H.L. (Finance code 001). CAPES also granted international internships to C.S.R‐S. (PDSE – 88881.624497/2021–01) and V.H.L (PDSE – 88881.623065/2021–01) at Texas A&M University.

## CONFLICT OF INTEREST STATEMENT

We declare that the authors have no conflict of interest.

## ETHICS STATEMENT

The study was conducted in accordance with the regulations of the Ethics Committee on Animal Use (CEUA) from Universidade Federal de Goiás (UFG) and Texas A&M University (TAMU) as described in Materials and Methods. Access to Brazilian genetic heritage was approved by the Genetic Heritage Management Council (CGen) of Brazil (SisGen protocol #A420934).

## Data Availability

The data that support the findings of this study are openly available in DRYAD at https://doi.org/10.5061/dryad.2547d7x5f (Ribeiro‐Silva et al., 2025).

## References

[mve70072-bib-0001] Adegoke, A. , Hanson, J. , Smith, R.C. & Karim, S. (2024) *Ehrlichia chaffeensis* co‐opts phagocytic hemocytes for systemic dissemination in the lone star tick, Amblyomma americanum. Journal of Innate Immunity, 16(1), 66–79. Available from: 10.1159/000535986 38142680 PMC10794049

[mve70072-bib-0002] Adegoke, A. , Ribeiro, J.M.C. , Brown, S. , Smith, R.C. & Karim, S. (2023) *Rickettsia parkeri* hijacks tick hemocytes to manipulate cellular and humoral transcriptional responses. Frontiers in Immunology, 14, 1094326. Available from: 10.3389/fimmu.2023.1094326 36845157 PMC9950277

[mve70072-bib-0003] Adegoke, A. , Ribeiro, J.M.C. , Smith, R.C. & Karim, S. (2024) Tick innate immune responses to hematophagy and *Ehrlichia* infection at single‐cell resolution. Frontiers in Immunology, 14, 1305976. Available from: 10.3389/fimmu.2023.1305976 38274813 PMC10808623

[mve70072-bib-0069] Adobe Inc. (2023) Adobe illustrator (version 27.6.1). San Jose, CA, USA: Adobe Inc. https://www.adobe.com/illustrator.

[mve70072-bib-0004] Alberdi, P. , Cabezas‐Cruz, A. , Prados, P.E. , Rayo, M.V. , Artigas‐Jerónimo, S. & de la Fuente, J. (2019) The redox metabolic pathways function to limit *Anaplasma phagocytophilum* infection and multiplication while preserving fitness in tick vector cells. Scientific Reports, 9(1), 13236. Available from: 10.1038/s41598-019-49766-x 31520000 PMC6744499

[mve70072-bib-0005] Araújo, R.P. , Navarro, M.B.M.A. & Cardoso, T.A.O. (2015) Spotted fever in Brazil: mortality study for epidemiological surveillance. Cadernos Saúde Coletiva, 23(4), 354–361. Available from: 10.1590/1414-462X201500040094

[mve70072-bib-0006] Arnhold, E. (2013) Package in the R environment for analysis of variance and complementary analyses. Brazilian Journal of Veterinary Research and Animal Science, 50(6), 488. Available from: 10.11606/issn.1678-4456.v50i6p488-492

[mve70072-bib-0007] Bahiense, T.C. , Fernandes, É.K.K. & Bittencourt, V.R.E.P. (2006) Compatibility of the fungus *Metarhizium anisopliae* and deltamethrin to control a resistant strain of *Boophilus microplus* tick. Veterinary Parasitology, 141(3–4), 319–324. Available from: 10.1016/j.vetpar.2006.05.011 16815637

[mve70072-bib-0008] Barré, N. , Li, A.Y. , Miller, R.J. , Gaïa, H. , Delathière, J.‐M. , Davey, R.B. et al. (2008) In vitro and in vivo evaluation of deltamethrin and amitraz mixtures for the control of *Rhipicephalus* (*Boophilus*) *microplus* (Acari: Ixodidae) in New Caledonia. Veterinary Parasitology, 155(1–2), 110–119. Available from: 10.1016/j.vetpar.2008.04.016 18565679

[mve70072-bib-0071] Blanco, L.A.A. , Crispim, J.S. , Fernandes, K.M. , Oliveira, L.L. , Pereira, M.F. , Bazzolli, D.M.S. et al. (2017) Differential cellular immune response of galleria mellonella to Actinobacillus pleuropneumoniae. Cell and Tissue Research, 370, 153–168. Available from: 10.1007/s00441-017-2653-5 28687931

[mve70072-bib-0009] Brinton, L.P. & Burgdorfer, W. (1971) Fine structure of normal hemocytes in *Dermacentor andersoni* stiles (Acari: Ixodidae). Journal of Parasitology, 57(5), 1110–1127. Available from: 10.2307/3277874 4332374

[mve70072-bib-0010] Cardoso, E.R.N. , Carvalho, S.F. , Dias, S.A. , Santos, R.A. , Tavares, M.A. , Neves, L.C. et al. (2023) Susceptibility of *Amblyomma sculptum*, vector of *rickettsia rickettsii*, ticks from a national park and an experimental farm to different synthetic acaricides. Pathogens, 12(11), 1304. Available from: 10.3390/pathogens12111304 38003769 PMC10675591

[mve70072-bib-0011] Carneiro, M.E. & Daemon, E. (2002) Estudo comparativo do aspecto da hemolinfa de algumas espécies de carrapatos (Acari, Ixodidae): descrição da variação hemocitária de adultos de *Amblyomma cajennense* (Fabricius) Koch. Revista Brasileira de Zoologia, 19(1), 171–175. Available from: 10.1590/s0101-81752002000500011

[mve70072-bib-0012] Ceraul, S.M. , Sonenshine, D.E. & Hynes, W.L. (2002) Resistance of the tick *Dermacentor variabilis* (Acari: Ixodidae) following challenge with the bacterium *Escherichia coli* (Enterobacteriales: Enterobacteriaceae). Journal of Medical Entomology, 39(2), 376–383. Available from: 10.1603/0022-2585-39.2.376 11931039

[mve70072-bib-0013] Chandler, D. , Davidson, G. , Pell, J.K. , Ball, B.V. , Shaw, K. & Sunderland, K.D. (2000) Fungal biocontrol of Acari. Biocontrol Science and Technology, 10(4), 357–384. Available from: 10.1080/09583150050114972

[mve70072-bib-0014] Cho, Y. & Cho, S. (2019) Hemocyte‐hemocyte adhesion by granulocytes is associated with cellular immunity in the cricket, Gryllus bimaculatus. Scientific Reports, 9(1), 18066. Available from: 10.1038/s41598-019-54484-5 31792279 PMC6889498

[mve70072-bib-0015] Corrêa, T.A. , Fiorotti, J. , Mesquita, E. , Meirelles, L.N. , Camargo, M.G. , Coutinho‐Rodrigues, C.J.B. et al. (2021) How dopamine influences survival and cellular immune response of *Rhipicephalus microplus* inoculated with *Metarhizium anisopliae* . Journal of Fungi, 7(11), 950. Available from: 10.3390/jof7110950 34829237 PMC8622812

[mve70072-bib-0016] Damasceno, I.A.M. & Guerra, R. (2018) *Coxiella burnetii* and Q fever in Brazil: a public health issue. Ciência & Saúde Coletiva, 23(12), 4231–4239. Available from: 10.1590/1413-812320182312.27772016 30540006

[mve70072-bib-0017] Eggenberger, L.R. , Lamoreaux, W.J. & Coons, L.B. (1990) Hemocytic encapsulation of implants in the tick *Dermacentor variabilis* . Experimental & Applied Acarology, 9(3–4), 279–287. Available from: 10.1007/bf01193434 2261820

[mve70072-bib-0018] Evans, A. , Madder, M. , Fourie, J. , Halos, L. , Kumsa, B. , Kimbita, E. et al. (2024) Acaricide resistance status of livestock ticks from east and West Africa and in vivo efficacy of acaricides to control them. International Journal for Parasitology: Drugs and Drug Resistance, 25, 100541. Available from: 10.1016/j.ijpddr.2024.100541 38761529 PMC11133915

[mve70072-bib-0019] Fan, J. , Wu, L. , Fan, X. , Li, Z. , Yuan, G. , Bi, Y. et al. (2025) Effects of *Beauveria bassiana* on immune responses in the silkworm, *Bombyx mori*: focus on haemocyte dynamics. Journal of Invertebrate Pathology, 213, 108424. Available from: 10.1016/j.jip.2025.108424 40825517

[mve70072-bib-0020] Feitosa, A.P.S. , Alves, L.C. , Chaves, M.M. , Veras, D.L. , Silva, E.M. , Aliança, A.S.S. et al. (2015) Hemocytes of *Rhipicephalus sanguineus* (Acari: Ixodidae): characterization, population abundance, and ultrastructural changes following challenge with *leishmania infantum* . Journal of Medical Entomology, 52(6), 1193–1202. Available from: 10.1093/jme/tjv125 26336264

[mve70072-bib-0021] Feitosa, A.P.S. , Chaves, M.M. , Veras, D.L. , Deus, D.M.V. , Portela‐Junior, N.C. , Araújo, A.R. et al. (2018) Assessing the cellular and humoral immune response in *Rhipicephalus sanguineus* sensu lato (Acari: Ixodidae) infected with *leishmania infantum* (Nicolle, 1908). Ticks and Tick‐borne Diseases, 9(6), 1421–1430. Available from: 10.1016/j.ttbdis.2018.06.007 30207274

[mve70072-bib-0022] Fernandes, É.K.K. & Bittencourt, V.R.E.P. (2008) Entomopathogenic fungi against south American tick species. Experimental and Applied Acarology, 46(1–4), 71–93. Available from: 10.1007/s10493-008-9161-y 18563593

[mve70072-bib-0072] Fiorotti, J. , Camargo, M.G. , Coutinho‐Rodrigues, C.J.B. , Marciano, A.F. , Freitas, M.C. , Silva, E.M. et al. (2018) Rhipicephalus microplus infected by Metarhizium: unveiling hemocyte quantification, GFP‐fungi virulence, and ovary infection. Parasitology Research, 117, 1847–1856. Available from: 10.1007/s00436-018-5874-y 29700639

[mve70072-bib-0023] Fiorotti, J. , Menna‐Barreto, R.F.S. , Gôlo, P.S. , Balduino, J. , Bitencourt, R.B. , Spadacci‐Morena, D.D. et al. (2019) Ultrastructural and cytotoxic effects of *Metarhizium robertsii* infection on *Rhipicephalus microplus* hemocytes. Frontiers in Physiology, 10, 654. Available from: 10.3389/fphys.2019.00654 31191351 PMC6548823

[mve70072-bib-0024] Fiorotti, J. , Urbanová, V. , Gôlo, P.S. , Bittencourt, V.R.E.P. & Kopácek, P. (2022) The role of complement in the tick cellular immune defense against the entomopathogenic fungus *Metarhizium robertsii* . Developmental and Comparative Immunology, 126, 104234. Available from: 10.1016/j.dci.2021.104234 34450130

[mve70072-bib-0025] Fisher, R.C. & Ganesalingam, V.K. (1970) Changes in the composition of host haemolymph after attack by an insect parasitoid. Nature, 227(5254), 191–192. Available from: 10.1038/227191a0 5428415

[mve70072-bib-0026] Fogaça, A.C. , Sousa, G. , Pavanelo, D.B. , Esteves, E. , Martins, L.A. , Urbanová, V. et al. (2021) Tick immune system: what is known, the interconnections, the gaps, and the challenges. Frontiers in Immunology, 12, 628054. Available from: 10.3389/fimmu.2021.628054 33737931 PMC7962413

[mve70072-bib-0027] Freitas, M.C. , Coutinho‐Rodrigues, C.J.B. , Perinotto, W.M.S. , Nogueira, M.R.S. , Chagas, T.T. , Marciano, A.F. et al. (2015) Quantificação de hemócitos em fêmeas ingurgitadas de *Rhipicephalus microplus* infectadas por *Beauveria bassiana* s.l. Revista Brasileira de Medicina Veterinária, 37(1), 63–70.

[mve70072-bib-0028] Gindin, G. , Samish, M. , Zangi, G. , Mishoutchenko, A. & Glazer, I. (2002) The susceptibility of different species and stages of ticks to entomopathogenic fungi. Experimental & Applied Acarology, 28(1–4), 283–288. Available from: 10.1023/a:1025379307255 14570142

[mve70072-bib-0029] Giulianini, P.G. , Bertolo, F. , Battistella, S. & Amirante, G.A. (2003) Ultrastructure of the hemocytes of *Cetonischema aeruginosa* larvae (coleoptera, Scarabaeidae): involvement of both granulocytes and oenocytoids in in vivo phagocytosis. Tissue and Cell, 35(4), 243–251. Available from: 10.1016/s0040-8166(03)00037-5 12921707

[mve70072-bib-0030] Gunnarsson, S.G.S. (1988) Infection of *Schistocerca gregaria* by the fungus *Metarhizium anisopliae*: cellular reactions in the integument studied by scanning electron and light microscopy. Journal of Invertebrate Pathology, 52(1), 9–17. Available from: 10.1016/0022-2011(88)90096-1

[mve70072-bib-0031] Hartelt, K. , Wurst, E. , Collatz, J. , Zimmermann, G. , Kleespies, R.G. , Oehme, R.M. et al. (2008) Biological control of the tick *Ixodes ricinus* with entomopathogenic fungi and nematodes: preliminary results from laboratory experiments. International Journal of Medical Microbiology, 298(1), 314–320. Available from: 10.1016/j.ijmm.2007.10.003

[mve70072-bib-0032] Hillyer, J.F. , Schmidt, S.L. & Christensen, B.M. (2003) Hemocyte‐mediated phagocytosis and melanization in the mosquito *Armigeres subalbatus* following immune challenge by bacteria. Cell and Tissue Research, 313(1), 117–127. Available from: 10.1007/s00441-003-0744-y 12838409

[mve70072-bib-0033] Kaplan, Z.D. , Richardson, E.A. , Taylor, C.E. , Kaufman, P.E. & Weeks, E.N.I. (2022) Determination of the discriminating concentration towards permethrin for surveying resistance in *Amblyomma americanum* . Journal of Medical Entomology, 59(3), 922–929. Available from: 10.1093/jme/tjac029 35323944 PMC9113138

[mve70072-bib-0034] King, J.G. & Hillyer, J.F. (2012) Infection‐induced interaction between the mosquito circulatory and immune systems. PLoS Pathogens, 8(11), e1003058. Available from: 10.1371/journal.ppat.1003058 23209421 PMC3510235

[mve70072-bib-0035] Labruna, M.B. , Leite, R.C. , Gobesso, A.A.O. , Gennari, S.M. & Kasai, N. (2004) Controle estratégico do carrapato *Amblyomma cajennense* em eqüinos. Ciência Rural, 34(1), 195–200. Available from: 10.1590/S0103-84782004000100030

[mve70072-bib-0036] Labruna, M.B. & Mattar, V.S. (2011) Rickettsioses in Latin America, Caribbean, Spain and Portugal. Revista MVZ Cordoba, 16(2), 2435–2457. Available from: 10.21897/rmvz.282

[mve70072-bib-0037] Lavine, M.D. & Strand, M.R. (2002) Insect hemocytes and their role in immunity. Insect Biochemistry and Molecular Biology, 32(10), 1295–1309. Available from: 10.1016/s0965-1748(02)00092-9 12225920

[mve70072-bib-0038] Leal‐Galvan, B. , Harvey, C. , Thomas, D. , Saelao, P. & Oliva Chavez, A.S. (2022) Isolation of microRNAs from tick ex vivo salivary gland cultures and extracellular vesicles. Journal of Visualized Experiments, 6(182), e63618. Available from: 10.3791/63618 35467650

[mve70072-bib-0039] Ling, E. & Yu, X.‐Q. (2005) Prophenoloxidase binds to the surface of hemocytes and is involved in hemocyte melanization in *Manduca sexta* . Insect Biochemistry and Molecular Biology, 35(12), 1356–1366. Available from: 10.1016/j.ibmb.2005.08.007 16291091

[mve70072-bib-0040] Lu, A. , Zhang, Q. , Zhang, J. , Yang, B. , Wu, K. , Xie, W. et al. (2014) Insect prophenoloxidase: the view beyond immunity. Frontiers in Physiology, 5, 1–16. Available from: 10.3389/fphys.2014.00252 25071597 PMC4092376

[mve70072-bib-0041] Lu, H.J. & St. Leger, R.J. (2016) Insect immunity to entomopathogenic fungi. Advances in Genetics, 94, 251–285. Available from: 10.1016/bs.adgen.2015.11.002 27131327

[mve70072-bib-0042] Martins, L.A. , Malossi, C.D. , Galletti, M.F.B.M. , Ribeiro, J.M. , Fujita, A. , Esteves, E. et al. (2019) The transcriptome of the salivary glands of *Amblyomma aureolatum* reveals the antimicrobial peptide microplusin as an important factor for the tick protection against *rickettsia rickettsii* infection. Frontiers in Physiology, 10, 529. Available from: 10.3389/fphys.2019.00529 31130872 PMC6509419

[mve70072-bib-0043] Massard, C. & Fonseca, A. (2004) Carrapatos e doenças transmitidas comuns ao homem e aos animais. Hora Veterinaria, 135(1), 15–23.

[mve70072-bib-0044] Mello, C.B. , Garcia, E.S. , Ratcliffe, N.A. & Azambuja, P. (1995) *Trypanosoma cruzi* and *Trypanosoma rangeli*: interplay with hemolymph components of *Rhodnius prolixus* . Journal of Invertebrate Pathology, 65(3), 261–268. Available from: 10.1006/jipa.1995.1040 7745280

[mve70072-bib-0045] Ment, D. , Churchill, A.C.L. , Gindin, G. , Belausov, E. , Glazer, I. , Rehner, S.A. et al. (2012) Resistant ticks inhibit *Metarhizium* infection prior to haemocoel invasion by reducing fungal viability on the cuticle surface. Environmental Microbiology, 14(6), 1570–1583. Available from: 10.1111/j.1462-2920.2012.02747.x 22507442

[mve70072-bib-0046] Mixson, T.R. , Campbell, S.B. , Gill, J.B. , Ginsberg, H.S. , Reichard, M.V. , Schulze, T.L. et al. (2006) Prevalence of *Ehrlichia*, *borrelia*, and *Rickettsial* agents in *Amblyomma americanum* (Acari: Ixodidae) collected from nine states. Journal of Medical Entomology, 43(6), 1261–1268. Available from: 10.1603/0022-2585(2006)43[1261:poebar]2.0.co;2 17162962

[mve70072-bib-0047] Miziara, C. , Serrano, V. & Yoshinari, N. (2018) Passage of *borrelia burgdorferi* through diverse ixodid hard ticks causes distinct diseases: Lyme borreliosis and Baggio‐Yoshinari syndrome. Clinics, 73, e394. Available from: 10.6061/clinics/2018/e394 30462754 PMC6218955

[mve70072-bib-0068] R Core Team . (2016) R: a language and environment for statistical computing. Vienna, Austria: R Foundation for Statistical Computing. https://www.R-project.org/

[mve70072-bib-0048] Reis, A.A.L. , Avelar, B.R. , Rocha, M.B.S. , Borges, D.A. , Campos, D.R. , Fiorotti, J. et al. (2024) Ultrastructural characterization and quantification of hemocytes in engorged female *Amblyomma sculptum* ticks. Ticks and Tick‐borne Diseases, 15(3), 102312. Available from: 10.1016/j.ttbdis.2024.102312 38277717

[mve70072-bib-0049] Ribeiro, V.L.S. , Santos, J.C. , Bordignon, S.A.L. , Apel, M.A. , Henriques, A.T. & Poser, G.L. (2010) Acaricidal properties of the essential oil from *Hesperozygis ringens* (Lamiaceae) on the cattle tick *Rhipicephalus* (*Boophilus*) *microplus* . Bioresource Technology, 101(7), 2506–2509. Available from: 10.1016/j.biortech.2009.11.016 19954969

[mve70072-bib-0050] Ribeiro‐Silva, C.S. , Lima, V.H. , Pinto, S.M.N. , Sandes, G.F. , Costa, V.H.R. & Mulenga, A. (2025) Cellular immune response of *Amblyomma sculptum* and *Amblyomma americanum* to entomopathogenic fungi: Implications for biological tick control. Dryad 10.5061/dryad.2547d7x5f PMC13436477 41964202

[mve70072-bib-0051] Ribeiro‐Silva, C.S. , Muniz, E.R. , Lima, V.H. , Bernardo, C.C. , Arruda, W. , Castro, R.N. et al. (2022) Cuticular lipids as a first barrier defending ixodid ticks against fungal infection. Journal of Fungi, 8, 1177. Available from: 10.3390/jof8111177 36354944 PMC9698823

[mve70072-bib-0052] Rosa, R.D. , Capelli‐Peixoto, J. , Mesquita, R.D. , Kalil, S.P. , Pohl, P.C. , Braz, G.R. et al. (2016) Exploring the immune signalling pathway‐related genes of the cattle tick *Rhipicephalus microplus*: from molecular characterization to transcriptional profile upon microbial challenge. Developmental and Comparative Immunology, 59, 1–14. Available from: 10.1016/j.dci.2015.12.018 26724380

[mve70072-bib-0053] Rozental, T. , Bustamante, M.C. , Amorim, M. , Serra‐Freire, N.M. & Lemos, E.R.S. (2002) Evidence of spotted fever group rickettsiae in state of Rio de Janeiro, Brazil. Revista do Instituto de Medicina Tropical de São Paulo, 44(3), 155–158. Available from: 10.1590/s0036-46652002000300008 12163909

[mve70072-bib-0054] Silva, S.B. , Savastano, G. & Bittencourt, V.R.E.P. (2006) Tipos celulares envolvidos na resposta imune de fêmeas ingurgitadas de *Boophilus microplus* inoculados em *Metarhizium anisopliae* e *Penicillium* sp. Revista Brasileira de Parasitologia Veterinária, 15(3), 128–131. 16978479

[mve70072-bib-0055] Solyman, M. , Brayton, K.A. , Shaw, D.K. , Omsland, A. , McGeehan, S. , Scoles, G.A. et al. (2021) Predicted iron metabolism genes in hard ticks and their response to iron reduction in *Dermacentor andersoni* cells. Ticks and Tick‐borne Diseases, 12(1), 101584. Available from: 10.1016/j.ttbdis.2020.101584 33059171

[mve70072-bib-0056] Sonenshine, D.E. & Hynes, W.L. (2008) Molecular characterization and related aspects of the innate immune response in ticks. Frontiers in Bioscience, 13(18), 7046–7063. Available from: 10.2741/3209 18508715

[mve70072-bib-0057] Sonenshine, D.E. & Roe, R.M. (2014) Biology of ticks. New York: Oxford University Press.

[mve70072-bib-0058] Sousa, L.A.D. , Pires Júnior, H.B. , Soares, S.F. , Ferri, P.H. , Ribas, P. , Lima, E.M. et al. (2011) Potential synergistic effect of *Melia azedarach* fruit extract and *Beauveria bassiana* in the control of *Rhipicephalus* (*Boophilus*) *microplus* (Acari: Ixodidae) in cattle infestations. Veterinary Parasitology, 175(3–4), 320–324. Available from: 10.1016/j.vetpar.2010.10.012 21055878

[mve70072-bib-0059] Strand, M.R. (2008) The insect cellular immune response. Insect Science, 15(1), 1–14. Available from: 10.1111/j.1744-7917.2008.00183.x

[mve70072-bib-0060] Stromdahl, E.Y. , Williamson, P. , Kollars, T.M. , Evans, S.R. , Barry, R.K. , Vince, M.A. et al. (2003) Evidence of *borrelia lonestari* DNA in *Amblyomma americanum* (Acari: Ixodidae) removed from humans. Journal of Clinical Microbiology, 41(12), 5557–5562. Available from: 10.1128/jcm.41.12.5557-5562.2003 14662940 PMC308998

[mve70072-bib-0061] Sullivan, C.F. , Parker, B.L. & Skinner, M. (2022) A review of commercial *Metarhizium*‐ and *Beauveria*‐based biopesticides for the biological control of ticks in the USA. Insects, 13(3), 260. Available from: 10.3390/insects13030260 35323558 PMC8952794

[mve70072-bib-0062] Szczotko, M. , Antunes, S. , Domingos, A. , Kubiak, K. & Dmitryjuk, M. (2024) Tick‐borne pathogens and defensin genes expression: a closer look at *Ixodes ricinus* and *Dermacentor reticulatus* . Developmental and Comparative Immunology, 160, 105231. Available from: 10.1016/j.dci.2024.105231 39043336

[mve70072-bib-0063] Tanaka, T. , Kawano, S. , Nakao, S. , Umemiya‐Shirafuji, R. , Rahman, M.M. , Boldbaatar, D . et al. (2010) The identification and characterization of lysozyme from the hard tick *Haemaphysalis longicornis* . Ticks and Tick‐borne Diseases, 1(4), 178–185. Available from: 10.1016/j.ttbdis.2010.09.001 21771526

[mve70072-bib-0064] Valencia, J.W.A. , Bustamante, A.L.G. , Jiménez, A.V. & Grossi‐de‐Sá, M.F. (2011) Cytotoxic activity of fungal metabolites from the pathogenic fungus *Beauveria bassiana*: an intraspecific evaluation of beauvericin production. Current Microbiology, 63(3), 306–312. Available from: 10.1007/s00284-011-9977-2 21761220

[mve70072-bib-0065] Wiegand, C. , Levin, D. , Gillespie, J.P. , Willott, E. , Kanost, M.R. & Trenczek, T. (2000) Monoclonal antibody MS13 identifies a plasmatocyte membrane protein and inhibits encapsulation and spreading reactions of *Manduca sexta* hemocytes. Archives of Insect Biochemistry and Physiology, 45(3), 95–108. Available from: 10.1002/1520-6327(200011)45:3<95::aid-arch1>3.0.co;2-0 11169749

[mve70072-bib-0066] Wrońska, A.K. , Kaczmarek, A. , Kazek, M. & Boguś, M.I. (2022) Infection of *Galleria mellonella* (lepidoptera) larvae with the entomopathogenic fungus *Conidiobolus coronatus* (Entomophthorales) induces apoptosis of hemocytes and affects the concentration of eicosanoids in the hemolymph. Frontiers in Physiology, 12, 774086. Available from: 10.3389/fphys.2021.774086 35069239 PMC8769874

[mve70072-bib-0067] Zibaee, A. , Bandani, A.R. , Talaei‐Hassanlouei, R. & Malagoli, D. (2011) Cellular immune reactions of the sunn pest, *Eurygaster integriceps*, to the entomopathogenic fungus, *Beauveria bassiana* and its secondary metabolites. Journal of Insect Science, 11, 138. Available from: 10.1673/031.011.13801 22233481 PMC3391913

